# Effects of Physical Prehabilitation on the Dynamics of the Markers of Endothelial Function in Patients Undergoing Elective Coronary Bypass Surgery

**DOI:** 10.3390/jpm12030471

**Published:** 2022-03-15

**Authors:** Yulia Argunova, Ekaterina Belik, Olga Gruzdeva, Sergey Ivanov, Svetlana Pomeshkina, Olga Barbarash

**Affiliations:** Federal State Budgetary Scientific Institution “Research Institute for Complex Issues of Cardiovascular Diseases”, 6, Sosnovy Boulevard, 650002 Kemerovo, Russia; sionina.ev@mail.ru (E.B.); o_gruzdeva@mail.ru (O.G.); ivansv@kemcardio.ru (S.I.); swetlana.sap2@mail.ru (S.P.); olb61@mail.ru (O.B.)

**Keywords:** coronary heart disease, coronary artery bypass surgery, prehabilitation, rehabilitation, physical training, endothelial dysfunction

## Abstract

Our aim in this study was to evaluate the effect of physical training performed before CABG on the perioperative dynamics of the serum levels of asymmetric dimethylarginine (ADMA) and endothelin-1 (ET-1) of patients with stable coronary heart disease (CHD). Patients in the preoperative period were randomized into two groups: the training group (*n* = 43) underwent high-intensity treadmill training; the patients in the control group (*n* = 35) received no training before the procedure. The serum concentrations of ADMA and ET-1 were determined in the perioperative period, and the course of the early postoperative period was analyzed. In the training group, we found a significantly lower incidence of postoperative complications during hospital stays (*p* = 0.013). At the end of the training program, the ADMA levels were 1.8 times higher in the controls than in the training group (*p* = 0.001). We found that type 2 diabetes increased the probability of complications by 12 times (OR: 12.3; 95% CI: 1.24–121.5; *p* = 0.03), as well as elevating the concentration of ET-1 on the eve of surgery (OR: 10.7; 95% CI: 1.4–81.3; *p* = 0.02). Physical prehabilitation reduced the likelihood of complications nine times (OR: 0.11; 95% CI: 0.02–0.83; *p* = 0.03). The AUC was 0.851 ± 0.07 (95% CI: 0.71–0.98). The obtained results indicate the benefit of physical training during the prehabilitation stage since it can help to preserve endothelial function.

## 1. Introduction

Prehabilitation as a method of preparing a patient for cardiac surgery is a subject of active research. The relevance of this topic lies in the high frequency of intra- and postoperative complications, primarily associated with myocardial damage and myocardial infarction (MI) [1]. Cardiovascular surgery with cardiopulmonary bypass (CPB) is associated with myocardial ischemia and reperfusion, warranting the study and implementation of measures aimed at increasing the resistance of cardiomyocytes to ischemia and reperfusion. Cardioprotective effects are most actively studied in patients with coronary heart disease (CHD) undergoing elective coronary artery bypass surgery (CABG). Preconditioning cardiomyocytes to ischemic damage is one of the promising methods aimed at protecting cardiomyocytes from ischemic damage [2].

In our previously published studies, we described the safety and clinical effectiveness of physical training as a method to prepare a patient for CABG, as well as the effects of preconditioning cardiomyocytes, which manifest as improved myocardial perfusion in the postoperative period and better clinical outcomes from the intervention [3]. Other authors have also demonstrated the positive effects of prehabilitation, including an improvement in the functional statuses of patients, reductions in postoperative complications and lengths of stay in the intensive care unit, and improvements in quality of life [4,5]. However, the discussion regarding the mechanisms of the cardioprotective effects of prehabilitation during cardiac surgery continues to this day. The available literature data suggest that physical training, one of the effective tools for modulating endothelial function, can be used in the preoperative period in order to manage endothelial dysfunction following surgical trauma and the subsequent triggering of inflammatory responses [1,6].

Nitric oxide (NO), a pleiotropic signaling molecule with a large spectrum of cardioprotective effects, triggers this process. The amino acid L-arginine is an essential substrate for vascular NO formation. One of its methylated derivatives, asymmetric dimethylarginine (ADMA), competitively inhibits NO synthase, disrupting the binding of L-arginine to the enzyme. A number of experimental and clinical studies have shown that ADMA contributes to the adverse effects of cardiovascular risk factors: an increase in total peripheral resistance, an increase in blood pressure, and a decrease in myocardial contractility [7]. A number of studies have proven the unfavorable prognostic role of ADMA in patients at risk of developing cardiovascular pathology. In a meta-analysis of 22 prospective studies, Willeit et al. suggested an association between high baseline ADMA and subsequent adverse cardiovascular events [8]. Demirci et al. conducted a study revealing a higher concentration of serum ADMA in patients with stable coronary heart disease and the no-reflow phenomenon [9]. They linked the development of this phenomenon to inflammation, oxidative stress, platelet dysfunction, and coronary vasomotor disorders.

Endothelin-1 (ET-1) is another participant in the development and progression of endothelial dysfunction. ET-1 is a predictor of an unfavorable prognosis and a powerful vasoconstrictor produced mainly by endothelial cells in response to angiotensin II production, inflammation and vascular wall tension, which contributes to vascular remodeling [10,11]. According to the literature data, elevated ET-1 levels can be observed in patients with hypertension, MI, atherosclerosis, and CHD. Moreover, elevated ET-1 levels are associated with an increased risk of adverse cardiac events [12], suggesting that it plays an important role in various CVDs. Experimental data showed that oxidized low-density lipoproteins stimulated both mRNA expression and ET-1 production by endothelial cells, confirming its role in the progression of atherosclerosis [13]. In addition to regulating vascular tone, ET-1 participates in vascular remodeling, promoting smooth muscle proliferation, protein synthesis, cytokine production, and growth factors [14].

The aim of this study was to assess the effect of physical training performed before CABG on the clinical outcomes and perioperative dynamics of the serum levels of ADMA and ET-1 in patients with stable coronary heart disease.

## 2. Material and Methods

### 2.1. Patients

This prospective, single-center, randomized study was conducted at the Research Institute for Complex Issues of Cardiovascular Diseases, Kemerovo, Russia. The research protocol received approval from the Ethics Committee of the Research Institute. Before inclusion in the study, the patients provided written informed consent. The study included 78 male patients aged 45–70 years with stable coronary heart disease scheduled for elective CABG with CPB. Considering the strict exclusion criteria, only 78 patients were included in the study out of 556 patients who had undergone CABG during the screening period. The decision to offer CABG was determined by a multidisciplinary heart team consisting of a cardiologist, a cardiovascular surgeon, and an endovascular surgeon, who evaluated the clinical data and the coronary lesion. The exclusion criteria for the study were as follows:

- Severe concomitant diseases that prohibit stress testing and physical training (severe chronic obstructive pulmonary disease, acute inflammation, skeletal and muscular system pathologies, and post-stroke recrudescence);
- A combination of coronary heart disease and valvular heart defects, and a left ventricular (LV) aneurysm;
- Scheduled brachiocephalic arterial reconstruction;
- Severe arrhythmias and conduction disorders, as well as atrial fibrillation;
- Thrombophlebitis and varicose veins of lower extremities with chronic venous insufficiency (class 3–4);
- Lower-extremity atherosclerosis with chronic limb ischemia higher than stage IIA, and peripheral arterial reconstruction in medical history;
- Aneurysms and aortic dissection;
- Decompensated chronic heart failure (CHF);
- NYHA class IV angina pectoris and NYHA class III CHF (and higher);
- Uncontrolled arterial hypertension (AH);
- A left ventricular ejection fraction (LV EF) less than 40%;
- Acute coronary syndrome;
- Stroke in the past 6 months;
- Surgery in the past 6 months;
- Significant left main coronary artery stenosis.

### 2.2. Methods of Physical Training

Following inclusion, the patients were randomized into two groups according to the prehabilitation approach. Envelope-based randomization was used to divide the patients into groups. A research team member not involved with the study conducted this procedure. The patients in the training group (*n* = 43; age: 61.5 (55.0, 65.0) years) underwent drug therapy according to clinical recommendations, the correction of modifiable risk factors, and treatment for concomitant pathologies. The daily treadmill workout (a walking exercise with continuous hemodynamic and electrocardiogram monitoring) lasted 5–10 days prior to CABG. The average number of workouts was 8.0 (7.0, 10.0). The exercise intensity of the main period was calculated individually, based on the peak oxygen consumption (VO_2_ peak) that was determined via cardiopulmonary exercise testing (CPET). A step-by step protocol was used. The load was increased gradually every 3 min, with build-up workloads of 25 W until the submaximal heart rate or the test termination criteria was reached. Indications for terminating CPET were as follows: symptoms at maximal exercise that included muscle fatigue, exhaustion, extreme dyspnea, or light-headedness; tachyarrhythmias; fall in systolic pressure > 20 mm Hg from the highest value during the test; chest pain suggestive of ischemia or ischemic ECG changes; second- or third-degree heart block; signs of respiratory failure; hypertension (>250 mm Hg systolic; > 120 mm Hg diastolic); and severe desaturation: Spo2 ≤80% when accompanied by symptoms and signs of severe hypoxemia. The VO_2_ peak was determined over the last 30 s of peak load and used to select the training load.

The training course was conducted on the background of standard medical therapy, including angiotensin-converting enzyme inhibitors/angiotensin II receptor antagonists, beta-blockers, statins, and antiplatelet agents as well as therapeutic and respiratory gymnastics. The daily training session lasted 40 min. The session was performed in the morning, and included a 5 min warm-up and a 5 min cool-down period (walking at a speed of 2.5 km/h in both cases) and a 30 min training phase. The duration of the training phase could be reduced at the patient’s request. The modified Borg scale was used to assess the load level (the optimal load level was 12–15 points). The VO_2_ peak remained constant (80%). The workload of the main phase was calculated according to [15]:

(1) $$U=\frac{0.06\cdot {\mathrm{VO}}_{2}\mathrm{target}-0.21}{0.1+0.018\cdot \alpha }$$

where U is the treadmill speed (km/h), VO_2_ target is the target oxygen consumption (mL/kg/min), and α is the angle of the treadmill inclination in degrees.

VO_2_ target = VO_2_ peak × 80%

Preparing the patients of the control group (*n* = 35; age: 63.0 (56.0, 66.0) years) for the surgery did not involve any physical training. All the patients underwent direct myocardial revascularization with CPB.

### 2.3. Laboratory Methods

The serum ADMA and ET-1 levels were measured using enzyme-linked immunosorbent assays. These markers were determined in the preoperative period before the training (1st stage), at end of the training program on the eve of surgery (2nd stage), and in the postoperative period within 5–7 days (3rd stage) after the procedure. The serum concentrations were determined in accordance with the manufacturer-provided instructions (ADMA—Immundiagnostik AG, Bensheim, Germany; ET-1—R&D Systems Inc., Minneapolis, MN, USA). During the analysis, control and test samples were incubated in microwell plates with pre-coated monoclonal antibodies. Adding biotin-conjugated monoclonal antibodies resulted in the binding of ADMA and ET-1 to the antibodies. Following the incubation, the wells were washed to remove the unbound biotin conjugate. HRP-conjugated streptavidin was added to bind to the ADMA and ET-1 conjugated to the biotin. After the second incubation, the wells were washed to remove the unbound streptavidin conjugate. Next, the substrate solution was added, forming a blue enzyme–substrate complex. The color then changed to yellow with the addition of the acid. The optical density corresponded to the intensity of the produced color in the wells and was measured spectrophotometrically at a wavelength of 450 nm using a standard calibration curve. Reference values for healthy individuals were taken based on the test systems recommended by manufacturers (for ADMA, 0.29–0.63 mmol; for ET-1, 0.47–2.0 pg/mL).

### 2.4. Postoperative Assessment

In the postoperative period during the hospital stay, the frequency of the development and types of complications were assessed. The combined endpoint (the total number of complications) and the incidence of the most life-threatening conditions were evaluated: MI, stroke, arrhythmias (including atrial fibrillation), inotropic therapy for heart failure, hydrothorax and hydropericardium, pneumonia, multiple organ dysfunction syndrome (MODS), and wound complications.

### 2.5. Statistical Methods

Statistical analysis was carried out using the Statistica software Version 10.0 (Statsoft Inc., Tulsa, OK, USA) software and IBM SPSS Statistics software Version 26 (IBM Corporation, Armonk, NY, USA) and included the calculation of the absolute values and percentages, and the medians and interquartile ranges (Me (Q25, Q75)). Intergroup differences were evaluated using nonparametric methods due to the non-normal distribution of the data. Fisher’s exact test was used to compare two independent groups on a qualitative basis, the Mann–Whitney U-test was used to compare two independent groups on a quantitative basis, the Wilcoxon signed-rank test was used to compare two dependent groups on a quantitative basis, and the Friedman test was used to compare three dependent groups. The relationship of two quantitative features was evaluated using Spearman’s rank-order correlation to study the strength and direction of the correlation.

We analyzed the influence of clinical, anamnestic, laboratory, and intraoperative factors on the risk of postoperative complications using logistic regression analysis. Simultaneously, we evaluated the development of complications in the postoperative period as an independent variable (occurrence of complications was coded as 1; the absence of complications was coded as 0). Considering the available literature data, the following factors affecting the risk of postoperative complications were selected: diabetes mellitus (DM) and chronic diseases of the bronchopulmonary system, the group according to the approach to prehabilitation, the ADMA and ET-1 levels on the eve of surgery, and intraoperative parameters. Due to the strict inclusion and exclusion criteria and the homogeneity of the studied groups, factors such as age, BMI, and other concomitant diseases were not included in the regression equation. Wald’s statistic was used for variable selection. The presence and level of association were assessed using the odds ratio (OR) and a 95% confidence interval (CI). Statistical significance was set at *p* < 0.05.

## 3. Results

At the first stage of the assessment of the data, we performed a comparative analysis of the baseline clinical and anamnestic characteristics of patients and the course of the intraoperative period (Table 1).

The patients did not differ in characteristics during the preoperative period, regarding comorbidities and anamnestic data. The patients were comparable in preoperative medication therapy. The patients were also comparable in the main parameters during the intraoperative period, suggesting that these factors did not affect the results of the study. Moreover, there were no differences in the intraoperative anesthetic management of the patients.

We analyzed the data collected using instrumental and functional research methods (echocardiography and CPET) at the preoperative stage before the training (Table 2).

The patients of both groups had similar baseline values obtained through echo and CPET.

All the study patients survived throughout the hospital stay. Cardiac arrhythmias (mainly atrial fibrillation), the development of heart failure, and complications of the bronchopulmonary system (hydrothorax and pneumonia) were the prevailing postoperative complications (Table 3).

During rehabilitation after CABG, the training group showed a significantly lower incidence of postoperative complications during their hospital stay compared to the controls (*p* = 0.013).

**Figure 1:**
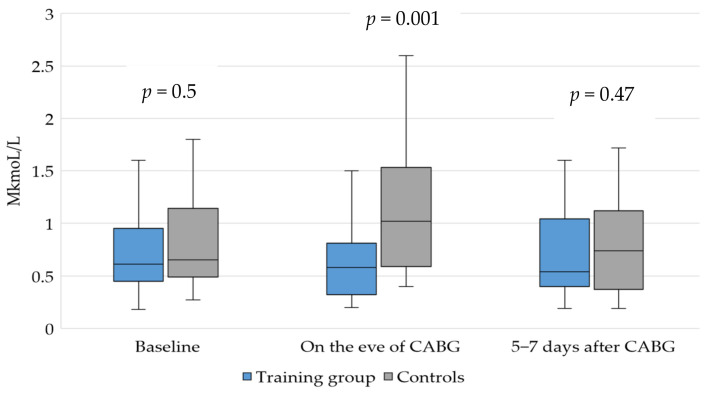
Changes in concentration of asymmetric dimethylarginine in the perioperative period for patients undergoing elective coronary bypass surgery according to the prehabilitation program.

Following that, we conducted an analysis of the concentration of ADMA in the perioperative period. A comparative assessment of the ADMA level in the patients before the start of the training program demonstrated the absence of significant intergroup differences. Some differences in the ADMA concentration between groups were found, but they were not statistically significant (Figure 1).

Performing exercise in the preoperative period did not significantly affect the serum ADMA. Higher ADMA levels were noted on the eve of CABG in the control group compared to the baseline values (1.6-fold increase, *p* > 0.05), and a statistically significant 1.8-fold increase in ADMA was noted in the controls compared to the training group at the end of the training program (*p* = 0.001). In the postoperative period, the concentration of ADMA in the training group did not change and remained within the reference values, whereas in the control group, the ADMA level returned to the baseline values. There were no intergroup differences in ADMA concentration after the procedure.

An inverse correlation between the amount of exercise performed during the preoperative stage and the ADMA at the end of the training program was found (*ρ* = −0.37, *p* = 0.035), reflecting the dose–response effects of physical activity.

Considering the known mechanism of ADMA clearance through renal excretion, to exclude the influence of this factor on the dynamics of ADMA in the groups, we evaluated the GFR in the perioperative period (Table 4).

We found no differences in these parameters; the eGFR remained within the reference values in all the patients at all stages of follow-up. Thus, the patients undergoing the prehabilitation program with physical training did not show statistically significant changes in serum ADMA concentration. However, the stability of the ADMA level was associated with exercise, as evidenced by the increase in ADMA concentration observed in the controls on the eve of surgery.

**Figure 2:**
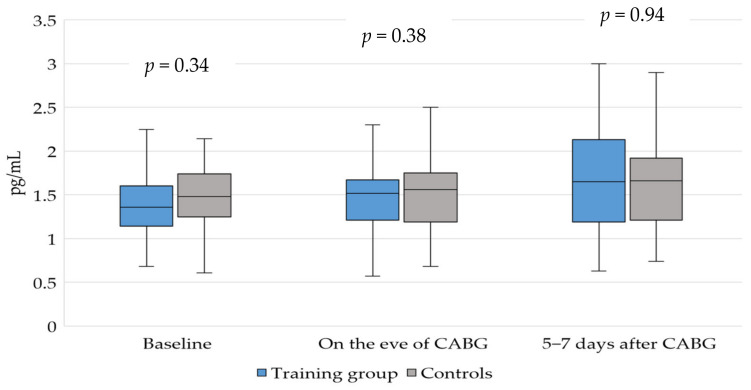
Changes in endothelin-1 concentration in the perioperative period for the patients undergoing elective coronary bypass surgery according to the approach to prehabilitation.

An analysis of the dynamics of the ET-1 concentration in the perioperative period demonstrated the absence of significant intergroup differences and the absence of reliable intragroup changes (Figure 2).

The correlation analysis of ADMA and ET-1 revealed a weak positive correlation between the concentrations of these markers on the eve of the surgery (*ρ* = 0.28, *p* = 0.04). Moreover, we found a moderate correlation during the assessment of the baseline concentrations (*ρ* = 0.35, *p* = 0.004); however, this was not detected in the postoperative period.

Following that, we developed a model for predicting the risk of complications in the early postoperative period using the binary logistic regression method. The obtained regression equation is presented below:

P = 1/(1 + e^−z^)

z = −2.8 + 2.51 × X (DM) + 2.37 × X (ET1) − 2.16 × X (group)

where P is the probability of developing complications in the early postoperative period, X (DM) is type 2 diabetes in medical history (0, absence; 1, presence), X (ET1) is the concentration of endothelin-1 on the eve of surgery (pg/mL), and X (group) is the group of patients according to the approach to prehabilitation (0, controls; 1, training group).

Thus, according to the obtained coefficients, the presence of type 2 diabetes increased the probability of complications in the early postoperative period 12 times (OR: 12.3; 95% CI: 1.24–121.5; *p* = 0.03). An increase in ET-1 concentration on the eve of surgery also increased the likelihood of complications (OR: 10.7; 95% CI: 1.4–81.3; *p* = 0.02). Exercising in the preoperative period, conversely, reduced the likelihood of complications nine times (OR: 0.11; 95% CI: 0.02–0.83; *p* = 0.03). This predictive model was statistically significant (*p* = 0.001), as characterized by a Nigelkirk coefficient of determination R^2^ of 0.44.

**Figure 3:**
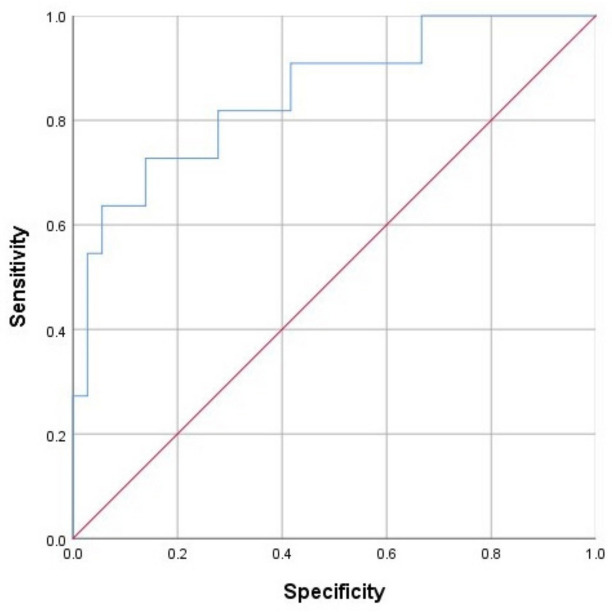
ROC curve characterizing the dependence of the probability of complications in the early postoperative period on the values of the P logistic function.

ROC analysis was performed to define a classification threshold and test the quality of the model (Figure 3).

The ROC AUC was 0.851 ± 0.07 (95% CI: 0.71–0.98). The cut-off value was determined at 0.17: for values 0.17 and higher, the probability of complications was high; for values below 0.17, the probability was low. The sensitivity and specificity of the predictive model at the selected threshold value were 81.8% and 72.2%, respectively.

Regarding the ET-1 concentration on the eve of surgery, we found that the ET-1 levels in patients with complications in the early postoperative period were significantly higher than those in patients without complications (1.73 (1.14, 2.44) pg/mL and 1.5 (1.19, 1.67) pg/mL, *p* = 0.01). At the same time, the ET-1 levels exceeded the upper threshold of the reference values in 22% of the patients with complications compared to 6.6% of the patients without complications (*p* = 0.07).

Therefore, factors such as type 2 diabetes, the ET-1 concentration on the eve of surgery, and physical exercise should be considered to adequately predict the course of the early postoperative period after CABG for male patients with stable coronary heart disease and without severe comorbidities.

## 4. Discussion

According to the obtained results, serum ADMA levels were not negatively affected by CABG in patients with or without physical prehabilitation, although the available literature data indicate an adverse effect of ischemic reperfusion injury following CABG or repeated episodes of ischemia, leading to numerous myocardial metabolic alterations, morphological changes in the myocardium, and elevated ADMA due to issues with ADMA degradation by dimethylarginindimethylaminohydrolase (DDAH) [16]. The results of the logistic regression analysis also show that the serum ADMA concentration is not associated with an increased risk of complications after CABG. In this study, despite being the fundamental initiator of the hyperproduction of pyroxynitrite and superoxide radicals, and the activation of oxidative stress [17], ADMA did not adversely affect the prognosis, due to the patients’ preserved renal function being at the level of the preoperative *GFR* in the postoperative period.

Being a marker of endothelial dysfunction, ADMA may manifest in ischemic reperfusion injury, in which endothelial dysfunction plays a key role in the genesis. Large amounts of inducible nitric oxide synthase (iNOS) increase the concentration of ADMA, which, in turn, acts as an endogenous NOS inhibitor and alters the NOS activity to favor the production of reactive oxygen species (ROS) [18]. The ADMA-induced uncoupling of iNOS in endothelial cells via the alteration of NO production to favor ROS is accompanied by the formation of a large number of superoxide radicals and toxic levels of peroxynitrite, which subsequently inhibit ADMA degradation by DDAH [19]. An excessive formation of peroxynitrite induces cell damage due to the irreversible nitration of protein tyrosine residues and indirect activation of matrix metalloproteinase 2 (MMP-2). MMP-2 is responsible for the intracellular degradation of contractile proteins, such as myosin light chain 1 (MLC1) [20]. MLC1 degradation by MMP-2 is enhanced during ischemia/reperfusion due to increased MLC1 phosphorylation [21]. Under physiological conditions, MMP-2 is necessary for the regulation of MLC levels [22]. Krzywonos-Zawadzka et al. showed that the increased iNOS production during ischemia/reperfusion causes an increase in ADMA production [16]. In clinical practice, ischemia and post-ischemic reperfusion are associated with significant loss of contractility and mechanical function of the myocardium due to the generation of oxidative stress, the subsequent increase in ROS, and the change in iNOS [23]. MMP-2 also destroys other contractile proteins, particularly MLC2 [20], troponin I (TnI) [24], and titin [25] in the ischemic myocardium, which can lead to disorders in myocardial contractility and heart failure.

Proinflammatory status in patients with coronary heart disease is strongly associated with the progression of atherosclerosis and the risk of adverse cardiac events. The results of previous studies demonstrated a positive relationship between circulating ADMA levels and IL-6 and CRP levels, indicating that inflammation and endothelial dysfunction are two parallel processes [26]. In vitro studies showed that ADMA induces TNF-α production via the ROS/NF-κB-dependent pathway [27]. The formation of ROS is an important initial event in inflammation: it inhibits the enzyme that degrades ADMA (DDAH), contributing to local myocardial and/or systemic ADMA accumulation, which, in turn, induces the formation of the proinflammatory mediators TNF-α and IL-8, activates the NF-κB-dependent pathway, and binds monocytes to endothelial cells [28]. The proper management of this condition with the help of prerehabilitation before surgery is another promising approach to reducing postoperative complications and improving the prognosis of patients.

Regular physical training can help to reduce the concentrations of ADMA in patients at high risk of developing cardiovascular diseases to the levels observed in healthy people. Mittermayer et al. determined the plasma ADMA, symmetric dimethylarginine (SDMA), and L-arginine levels in patients with type 1 diabetes who participated in a supervised aerobic exercise program for four months. According to the results of the study, after two and four months of exercise, the ADMA concentrations in the patients decreased to those seen in healthy persons compared to the ADMA levels before training began. However, 8 months after the cessation of the exercise program, the ADMA levels reverted to those observed before the start of training [29].

Another team of researchers proved that a short exercise program may be enough to reduce the level of ADMA. As part of the study, the researchers determined the plasma ADMA, SDMA, and L-arginine levels in patients with coronary heart disease undergoing CABG and PCI, before and after exercise (walking on a treadmill for 10 min). The obtained data revealed significant decreases in ADMA and SDMA and an increase in the substrate of endothelial NO (L-arginine) [30]. Hambrecht et al. [31] conducted a study that included patients with stable coronary heart disease and preserved LV EF, scheduled for CABG utilizing the left internal thoracic artery as a conduit. A four-week physical training program was conducted in the preoperative period, and the exercise intensity was determined using the maximum workload that would not induce chest pain syndrome. During the procedure, the biopsy from the left internal thoracic artery was examined. The authors found a significant increase in endothelium-dependent vasodilation due to the expression of endothelial NO synthase in the training group. Thus, the available literature data indicate the effectiveness of both short and long programs of physical training in reducing the circulating levels of ADMA. Notably, no such effect was observed in the present study. This may have been due to the cessation of the training program before the surgery and the short duration of the exercises in general. Nevertheless, even this short prehabilitation program proved its effectiveness in maintaining the physiological concentration of ADMA on the eve of CABG, which was not observed in controls.

The mechanism of ensuring the optimal expression and activity levels of DDAH, an enzyme involved in ADMA metabolism in vivo, also played a role in ensuring the stable serum ADMA level in the prehabilitation group. The increased renal blood flow during exercise ensures coordination between the work of DDAH, which is responsible for the degradation of more than 80% of ADMA and the clearance of ADMA in the kidneys [32]. Moreover, due to the direct stimulation of DDAH gene expression, it is activated during exercise [33]. Based on these data, we assume that DDAH activation occurred only in patients who received physical training before CABG, since this process was not observed in patients without prehabilitation. This assumption is supported by the results obtained by Ammar et al. in a study of the effect of physical exercises performed three times per week for ≥6 months in the predialysis period in patients with end-stage renal failure (predialytic 10–12 stretching cycles and 20–30 min of intradialytic pedaling cycling). The authors reported a moderate decrease in ADMA levels in persons with a higher physical activity level [34].

Supposedly, both the intensity and duration of exercise affect the degree of reduction in ADMA. These parameters have a hypothetical threshold below which the desirable clinical effect is not achieved [26], hence the need to calculate a workload specific to the patient and provide patient-centered care. This assumption is based on the results of studies demonstrating that each exercise session creates a circulating anti-inflammatory environment due to the myokines produced, expressed, and released by muscle fibers, which have paracrine, autocrine, and endocrine effects [35].

Although ET-1 is mainly produced by the vascular endothelium, other cells such as smooth muscle cells, fibroblasts, and macrophages are known sources of ET-1. ET-1 induces the production of proinflammatory cytokines. ET-1′s expression is inhibited by NO, prostacyclin, and atrial natriuretic peptide [36].

An increase in the ET-1 concentration was demonstrated in exercise-induced myocardial ischemia detected using CPET, suggesting that ET-1 is a marker of the severity of ischemia and not the ischemia itself. Li et al. studied the effect of simvastatin on exercise-induced myocardial ischemia in patients with stable angina. The results show that elevated ET-1 was associated with an increased risk of adverse cardiac events and the presence of angiographically confirmed coronary heart disease; additionally, an increase in this marker was observed both in non-Q-wave and in Q-wave MI, and was associated with the degree of myocardial ischemia [37]. Previous studies showed that an increased plasma ET-1 concentration is associated with the intensity of angina symptoms, suggesting that ET-1 may have an effect on the degree of myocardial ischemia [38]. ET-1 can also contribute to exercise intolerance in patients with heart failure by impairing vasodilation during exercise and can serve as a marker of the severity of exercise-induced ischemia [39].

The lack of changes in ET-1 concentration in this study can be explained by the short duration of the training program. The results obtained by other authors confirm this assumption. Davis et al., who studied the effect of the duration of a treadmill workout at an intensity of 70% VO_2_ max in 11 men, showed that a short-term training session did not change the concentration of plasma ET-1 immediately after training. However, an increase in ET-1 levels was observed after a long session; moreover, the changes were time-dependent [40]. Maeda et al., who studied the effect of eight-week physical exercises that lasted 1 h, 3–4 days per week, on ET-1 and NO concentrations in healthy young people, showed a significant decrease in the concentration of ET-1 (1.65 ± 0.14 vs. 1.23 ± 0.12 pg/mL, *p* < 0.05) and an increase in the NO concentration (30.69 ± 3.20 vs. 48.64 ± 8.16 mmol/L, *p* < 0.05) after training. Moreover, a significant negative correlation between the plasma concentrations of ET-1 and NO was revealed. However, the observed changes remained until the fourth week after the cessation of the training program, with the ET-1 returning to the baseline values on the eighth week after the cessation of training. Based on the results obtained, the authors suggested that constant training contributed to a decrease in ET-1 and an increase in NO production [41].

Mehrabi et al. comparatively assessed the effect of eight-week, low-intensity aerobic exercises on healthy people and patients with coronary heart disease. The results show that, throughout the study, no significant changes in plasma ET-1 concentration occurred. The authors concluded that there is a threshold in terms of exercise time or intensity for stimulating hormone secretion, with intensities and durations of exercise that fall below this stimulation threshold resulting in insignificant changes in the ET-1 concentration [42]. According to Naya et al., an increase in the concentration of serum ET-1 was a predictor of the development of coronary microvascular dysfunction in patients with stable coronary heart disease, which manifested as a coronary flow velocity reserve decrease in the nonoccluded vascular bed. At the same time, serum ET-1 was positively associated with the myocardial blood flow, estimated using positron emission tomography and pharmacological stress testing. According to the authors’ conclusions, these results support a pronounced decrease in coronary flow velocity reserve due to stress in patients with coronary heart disease and elevated ET-1 [43].

Considering the category of patients undergoing CABG, it is also necessary to take into account the drug therapy received by the patients. The absence of changes in the ET-1 concentration in the perioperative period and the significant intergroup differences in our study could have been a consequence of the influence of the prescribed drug therapy. The available data indicate the ability of beta-blockers [44], vasodilators [45], statins [37], and angiotensin-converting enzyme inhibitors [46] to reduce plasma ET-1 levels. Li et al. revealed that simvastatin rapidly reduced plasma ET-1 concentrations in patients with chronic coronary heart disease: two-week simvastatin therapy reduced ET-1 by 44%, whereas no changes in ET-1 concentration were detected in the placebo group [37]. Moreover, scientific data indicate the ability of hydrophilic statins, such as rosuvastatin, to significantly reduce serum ADMA levels [47].

### Study Limitations

This study is limited by its focus on a specific category of male CABG patients without significant comorbidity. Another limitation of the study is a small sample size. These issues mean that future research on this topic should be conducted.

## 5. Conclusions

The obtained results indicate the benefit of physical training at the stage of prehabilitation since it can help to preserve endothelial function and overcome vasospasms both on the eve of surgery and during the intraoperative period. However, we speculate that brief physical exercise before the procedure would not provide the desired effect, and that physical training should be continued after surgery as well. The results of this pilot study revealed certain patterns, but future research on this topic is still required.

## Figures and Tables

**Table 1:** Clinical, anamnestic, and intraoperative characteristics of the patients.

Parameters	Training Group (*n* = 43)	Controls (*n* = 35)	*p*
Age, years (Me (Q25, Q75))	61.5 (55, 65)	63.0 (56, 66)	0.43
Working patients, *n* (%)	16 (37.2)	12 (34.2)	0.79
BMI, kg/m^2^ (Me (Q25, Q75))	29.3 (25.9, 30.9)	28.6 (26.4, 31.6)	0.81
Smoking, *n* (%)	13 (30.2)	10 (28.5)	0.87
EuroScore (Me (Q25, Q75))	0.75 (0.6, 0.79)	0.84 (0.68, 0.9)	0.36
CHD duration, years (Me (Q25, Q75))	1.0 (0.5, 3.0)	1.0 (0.5, 6.0)	0.41
AH, *n* (%)	36 (83.7)	33 (94.2)	0.15
AH duration, years (Me (Q25, Q75))	4.5 (2.0, 10.0)	5.0 (3.0, 10.0)	0.56
AF, *n* (%)	2 (4.6)	2 (5.7)	0.82
Angina pectoris FC NYHA, *n* (%): 0-I	10 (23.2)	7 (20)	0.73
II	29 (67.4)	25 (71.4)	
III	4 (9.3)	3 (8.5)	
CHF FC NYHA, *n* (%): 0-I	1 (2.3)	0	0.36
II	42 (97.7)	35 (100)	
Prior myocardial infarction, *n* (%)	30 (69.7)	22 (62.8)	0.52
History of prior stroke, *n* (%)	3 (6.9)	2 (5.7)	0.83
DM in medical history, *n* (%)	9 (20.9)	7 (20)	0.92
Severity of coronary lesions, SYNTAX score (Me (Q25, Q75))	24.2 (18.5, 29.8)	23.8 (17.8, 28.3)	0.43
Operative time, min (Me (Q25, Q75))	200.0 (170.0, 220.0)	210.0 (180.0, 240.0)	0.68
Aortic clamping, min (Me (Q25, Q75))	52.5 (48.0, 59.5)	51.0 (46.0, 60.0)	0.36
CPB, min (Me (Q25, Q75))	78.5 (71.0, 89.0)	82.0 (73.0, 95)	0.27
Number of shunts, *n* (Me (Q25, Q75))	2.0 (2.0, 3.0)	2.0 (2.0, 3.0)	0.98
Cardioplegia infusion, *n* (Me (Q25, Q75))	2.0 (2.0, 3.0)	2.0 (2.0, 3.0)	0.98
Lowest body temperature (before CPB), °C (Me (Q25, Q75))	35.7 (35.3, 35.8)	35.7 (35.3, 35.7)	0.96
Lowest systolic BP, mm Hg (Me (Q25, Q75))	100.0 (93.0, 103.0)	99.0 (92.0, 105.0)	0.34
Intraoperative blood loss, mL (Me (Q25, Q75))	500.0 (400.0, 500.0)	500.0 (400.0, 500.0)	0.97
Total blood loss, mL (Me (Q25, Q75))	800.0 (650.0, 850.0)	800.0 (700.0, 950.0)	0.78

Note: AH—arterial hypertension; BP—blood pressure; CHD—coronary heart disease; CPB—cardiopulmonary bypass; BMI—body mass index; DM—diabetes mellitus; FC—functional class; and CHF—chronic heart failure.

**Table 2:** Echocardiography and cardiopulmonary exercise test results for the patients according to the approach to prehabilitation.

Parameters (Me (Q25, Q75))	Training Group (*n* = 43)	Controls (*n* = 35)	*p*
LV EF, %	63.0 (60.0, 67.0)	64.0 (61.0, 66.0)	0.96
LV EDV, mL	147.5 (130.0, 173.0)	147.0 (130.0, 180.0)	0.24
LV EDD, mL	54.0 (47.0, 74.0)	51.0 (44.0, 62.0)	0.23
LV ESV, cm	5.5 (5.2, 5.9)	5.5 (5.2, 6.0)	0.31
LV ESD, cm	3.6 (3.4, 4.1)	3.5 (3.3, 3.8)	0.47
VO_2_ peak, mL/kg/min	15.3 (13.4, 18.3)	15.7 (13.7, 17.1)	0.64
VO_2_ peak, %	59.0 (55.0, 70.0)	63.0 (54.0, 69.0)	0.78
AT, mL/kg/min	11.9 (10.3, 14.9)	12.5 (10.9, 15.7)	0.24
AT, %	47.5 (43.0, 59.0)	49.5 (44.0, 62.0)	0.15
Peak heart rate, bpm	110 (98, 126)	112 (97, 129)	0.57
Exercise tolerance, W	75.0 (75.0, 100.0)	87.5 (75.0, 100.0)	0.78

Note: AT—anaerobic threshold; LV EDV—end diastolic volume of the left ventricle; LV EDD—end diastolic dimension of the left ventricle; LV ESV—end systolic volume of the left ventricle; LV ESD—end systolic dimension of the left ventricle; LV EF—left ventricular ejection fraction; and VO_2_ peak—peak oxygen consumption.

**Table 3:** Postoperative complications of patients after coronary bypass surgery according to the prehabilitation program.

Parameters	Training Group (*n* = 43)	Controls (*n* = 35)	*p*
Combined endpoint, *n* (%)	5 (11.6)	13 (37)	0.013
Myocardial infarct, *n* (%)	0	1 (2.8)	0.44
Stroke, *n* (%)	1 (2.3)	1 (2.8)	0.56
Arrhythmias, *n* (%)	2 (4.6)	3 (8.5)	0.65
Heart failure, *n* (%)	2 (4.6)	6 (17)	0.13
Hydrothorax, *n* (%)	0	2 (5.7)	0.19
Hydropericardium, *n* (%)	0	0	-
Pneumonia, *n* (%)	0	2 (5.7)	0.19
MODS, *n* (%)	0	0	-
Wound complications, *n* (%)	0	0	-

**Table 4:** Estimates of glomerular filtration rate in the perioperative period for patients undergoing elective coronary bypass surgery according to the approach to prehabilitation.

eGFR (CKD-EPI), mL/min/1.73 m^2^ (Me (Q25, Q75))	Training Group (*n* = 43)	Controls (*n* = 35)	*p*
Baseline	81.0 (73.0, 92.0)	78.0 (69.0, 91.0)	0.43
On the eve of CABG	83.0 (74.0, 95.0)	82.0 (71.0, 92.0)	0.54
5–7 days after CABG	85.0 (75.0, 99.5)	85.0 (68.0, 97.0)	0.78

## Data Availability

Data sharing not applicable.
